# Draft Genome Sequence of *Jeotgalibacillus soli* DSM 23228, a Bacterium Isolated from Alkaline Sandy Soil

**DOI:** 10.1128/genomeA.00512-15

**Published:** 2015-05-21

**Authors:** Kian Mau Goh, Kok-Gan Chan, Amira Suriaty Yaakop, Chia Sing Chan, Robson Ee, Wen-Si Tan, Han Ming Gan

**Affiliations:** a Faculty of Biosciences and Medical Engineering, Universiti Teknologi Malaysia, Skudai, Johor, Malaysia; b Division of Genetics and Molecular Biology, Institute of Biological Sciences, Faculty of Science, University of Malaya, Kuala Lumpur, Malaysia; c Monash School of Science, Monash University Sunway Campus, Petaling Jaya, Selangor, Malaysia

## Abstract

*Jeotgalibacillus soli*, a bacterium capable of degrading *N*-acyl homoserine lactone, was isolated from a soil sample in Portugal. *J. soli* constitutes the only *Jeotgalibacillus* species isolated from a non-marine source. Here, the draft genome, several interesting glycosyl hydrolases, and its putative *N*-acyl homoserine lactonases are presented.

## GENOME ANNOUNCEMENT

*Jeotgalibacillus* is under explored halophilic genus of family *Planococcaceae*. The cell wall peptidoglycan of members of this genus is of the A1α type, linked directly through l-Lys. The major quinones of *Jeotgalibacillus* spp. are MK-7 and MK-8 (1). With the exception of *J. soli* DSM 23228, isolated from alkaline sandy soil, representatives of this genus are associated with marine sources or fermented seafood. *J. soli* has been identified as being strictly aerobic, oxidase- and catalase-positive, and positive for H_2_S production (2). Cells have single polar or subpolar flagella. *J. soli* is distinctive from other *Jeotgalibacillus* spp. in its limited tolerance to NaCl (maximum 9% [wt/vol]). Other species such as *J. alimentarius*, *J. salarius*, *J. malaysiensis*, and *J. campisalis*, for instance, are able to tolerate concentrations of 15 to 30% (wt/vol) (1, 3, 4).

Strain DSM 23228 was obtained from the Deutsche Sammlung von Mikroorganismen und Zellkulturen and its genome was sequenced using an Illumina MiSeq sequencer. An average coverage of 200-fold was obtained for the draft genome of 3,776,953 bp in 24 contigs with a *N*_50_ of 525,494. The *de novo* assembly was performed using SPAdes (5). Gene prediction was carried out using Glimmer 3.02 (6), tRNA prediction with tRNAscan-SE (7), and rRNA prediction with HMMER (8), while BLAST searches were performed against several databases including CatFam, COG, NCBI RefSeq, and SEED.

The G+C content of the *J. soli* genome is 39.7%. The total number of predicted genes is 3,938, and 5 rRNA and 78 tRNA genes were identified. Protein coding genes with predicted functions number 3,040, equivalent to approximately 80% of the total number of predicted genes. Of these, 968 sequences putatively code for catalytic enzymes. The ability of *J. soli* to use starch as a carbon source may be explained by the presence of enzymes (3 α-amylases and 2 pullulanases) that act on α-1,4 and α-1,6 glycosidic bonds. In addition, our detection of the key glycogen-degrading enzyme oligo-1,4-1,6-α-glucosidase is consistent with the capacity of *J. soli* to grow on glycogen. Although β-glucosidase activity was not observed using a standard API 50 CHB/*E* test (bioMérieux), a gene encoding this enzyme was identified in the *J. soli* genome. In addition, the *N*-acyl homoserine lactone (AHL) degradation capability of *J. soli* was validated using an AHL inactivation assay performed with *N*-hexanoyl-l-homoserine lactone and *N*-(3-oxohexanoyl)-l-homoserine lactone, and several putative *N*-acyl homoserine lactonases (9, 10) were identified in the genomic sequence. Based on the same assay, *J. alimentarius*, *J. salarius*, *J. malaysiensis*, and *J. campisalis* were found to be unable to degrade AHL.

### Nucleotide sequence accession numbers.

This whole-genome shotgun project has been deposited in DDBJ/EMBL/GenBank under the accession no. JXRP00000000. The version described in this paper is the first version, JXRP01000000.
